# Xenogeneic cross-circulation for physiological support and recovery of ex vivo human livers

**DOI:** 10.1097/HEP.0000000000000357

**Published:** 2023-03-30

**Authors:** Wei Kelly Wu, Rei Ukita, Yatrik J. Patel, Michael Cortelli, Vincent Q. Trinh, Ioannis A. Ziogas, Sean A. Francois, Meredith Mentz, Nancy L. Cardwell, Jennifer R. Talackine, William M. Grogan, John W. Stokes, Youngmin A. Lee, Jinho Kim, Sophoclis P. Alexopoulos, Matthew Bacchetta

**Affiliations:** 1 Division of Hepatobiliary Surgery and Liver Transplantation, Vanderbilt University Medical Center, Nashville, Tennessee, USA; 2 Department of Cardiac Surgery, Vanderbilt University Medical Center, Nashville, Tennessee, USA; 3 Section of Surgical Sciences, Vanderbilt University Medical Center, Nashville, Tennessee, USA; 4 Department of Biomedical Engineering, Stevens Institute of Technology, Hoboken, New Jersey, USA; 5 Department of Biomedical Engineering, Vanderbilt University; Nashville, Tennessee, USA

## Abstract

**Background and Aims:**

The scarcity of suitable donor livers highlights a continuing need for innovation to recover organs with reversible injuries in liver transplantation.

**Approach and Results:**

Explanted human donor livers (n = 5) declined for transplantation were supported using xenogeneic cross-circulation of whole blood between livers and xeno-support swine. Livers and swine were assessed over 24 hours of xeno-support. Livers maintained normal global appearance, uniform perfusion, and preservation of histologic and subcellular architecture. Oxygen consumption increased by 75% (*p* = 0.16). Lactate clearance increased from −0.4 ± 15.5% to 31.4 ± 19.0% (*p* = 0.02). Blinded histopathologic assessment demonstrated improved injury scores at 24 hours compared with 12 hours. Vascular integrity and vasoconstrictive function were preserved. Bile volume and cholangiocellular viability markers improved for all livers. Biliary structural integrity was maintained.

**Conclusions:**

Xenogeneic cross-circulation provided multisystem physiological regulation of ex vivo human livers that enabled functional rehabilitation, histopathologic recovery, and improvement of viability markers. We envision xenogeneic cross-circulation as a complementary technique to other organ-preservation technologies in the recovery of marginal donor livers or as a research tool in the development of advanced bioengineering and pharmacologic strategies for organ recovery and rehabilitation.

## Graphical abstract

**Figure:**
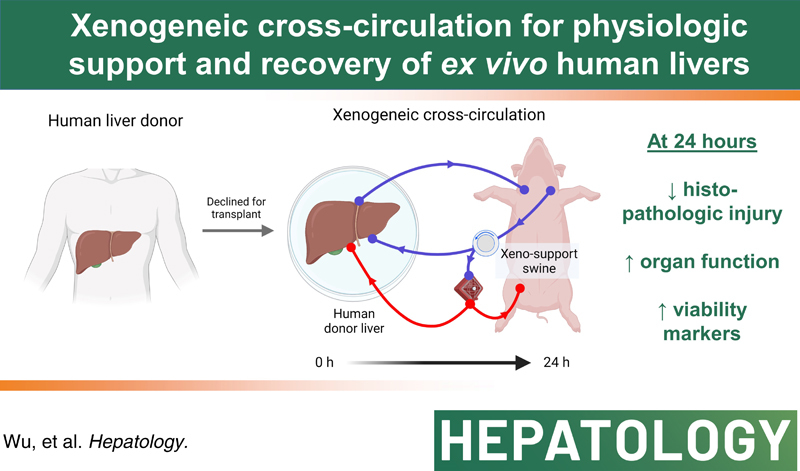

## INTRODUCTION

The scarcity of suitable donor organs remains one of the greatest challenges in the modern era of transplantation.^1^ Current limitations in organ salvage and rehabilitation impose significant barriers to liver transplantation and regenerative medicine.

Numerous strategies have been attempted to address the gap in organ supply and demand.^1^ Use of donors after cardiac death (DCD), older donors, or those with steatotic livers is associated with an increased risk of early allograft dysfunction and ischemic cholangiopathy.^2^ Split-liver transplantation is associated with increased technical complexity and post-transplant complications.^3^ Xenotransplantation of organs from genetically engineered swine has seen a wave of recent progress in kidney and heart transplantation^4,5^ but lags behind in liver transplantation.^6^

Currently, the most clinically used method for enhanced preservation of high-risk donor livers is by machine perfusion. Compared with static cold storage, machine perfusion allows continuous delivery of oxygen and nutrients, permits an opportunity to evaluate graft viability ex vivo, and is associated with decreased rates of biliary complications and early allograft dysfunction.^7,8^ A recent report described successful preservation of 6 discarded human donor livers for 7 days using an integrated machine perfusion system.^9^ Despite these remarkable feats, machine perfusion systems remain limited in their ability to recover many organs with severe injuries, possibly due to the absence of key processes that are only maintained in vivo, including multisystem synthetic function, neurohormonal signaling, and complex interorgan crosstalk.

A platform that can replicate a normal, physiological milieu may enable more robust ex vivo organ rehabilitation, repair, and investigation of advanced therapeutics. Our group developed a platform for the physiological maintenance of ex vivo swine livers using cross-circulation.^10^ This technique enabled functional organ preservation with improved histopathologic injury over 12 hours. In studies in the lung, our group also demonstrated that cross-circulation supported the functional recovery and multiday maintenance of ex vivo swine lungs,^11–14^ as well as the viability and functional recovery of unallocated human lungs using xenogeneic cross-circulation (XC).^15^

We hypothesize that, likewise, XC of ex vivo human livers could enable multisystem physiological regulation, preservation of tissue integrity, and functional organ rehabilitation. In this study, explanted human donor livers declined for clinical transplantation were placed on cross-circulation with swine xeno-support subjects. Throughout 24 hours of support, human livers were subject to longitudinal evaluations of metabolic and synthetic function, macro and micro-architectural integrity, cellular viability and injury, and immunologic activity and inflammatory response.

## METHODS

### Animals

Wild-type Yorkshire x Landrace swine (n = 5), 4–5 months of age, with a mean weight of 72 ± 11 kg (range, 55–84 kg) were used. The study was approved by the Institutional Animal Care and Use Committee at Vanderbilt University Medical Center and conducted in accordance with the US National Research Council of the National Academies “Guide for the Care and Use of Laboratory Animals, Eighth Edition”.

### Human donor livers

Donor livers (n = 5) declined for transplantation and consented for research use were procured in coordination with local organ procurement organizations under protocols approved by the Institutional Review Board at Vanderbilt University Medical Center and in accordance with the Declarations of Helsinki and Istanbul. Deidentified donor data were obtained through the United Network for Organ Sharing under approved protocols. Livers from donors with a history of HIV, active hepatitis *B* or *C* infection, or with anticipated cold ischemia time exceeding 24 hours were excluded from this study.

### Immunosuppression

**FIGURE 1:**
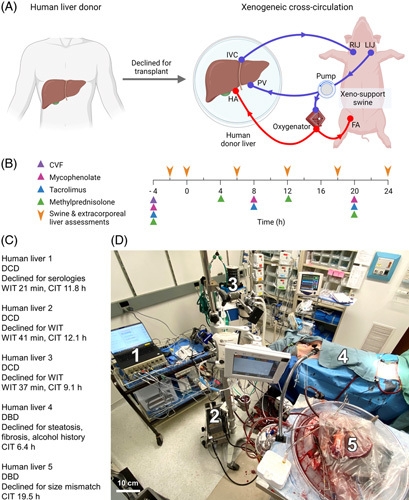
Maintenance of explanted human livers using XC. (A) Human donor livers declined for transplantation were procured in a standard manner, transported to the study site, and cannulated before initiation of cross-circulation with a xeno-support swine that was treated with an immunosuppression regimen. (B) Timeline for experimental assessments and administration of immunosuppression regimen over the course of 24 hours of XC. (C) Characteristics of the 5 human livers at the initiation of XC. (D) Experimental setup for XC procedures: (1) perfusion data acquisition and display; (2) pump and console; (3) host ventilator, anesthetic monitors, and infusions; (4) xeno-support swine; and (5) extracorporeal human liver. Abbreviations: CIT, cold ischemia time; CVF, cobra venom factor; DBD, donors after brain death; DCD, donor after cardiac death; FA, femoral artery; HA, hepatic artery; LIJ, left internal jugular vein; PV, portal vein; RIJ, right internal jugular vein; WIT, warm ischemia time; XC, xenogeneic cross-circulation.

To minimize the risk of rejection, an immunosuppression regimen informed by current practices in clinical transplantation, and described for use in xeno-support of human lungs, was used (Figure 1B).^14,15^ At 4 hours before cross-circulation, xeno-support swine were anesthetized, intubated, and administered cobra venom factor (1 mg; Sigma-Aldrich) to deplete complement activity.^16^ Intravenous diphenhydramine (50 mg; West Ward) and methylprednisolone (1 g; Pfizer) were administered to limit the inflammatory response associated with cobra venom factor. Intravenous tacrolimus (5 mg; Astellas) and mycophenolate (500 mg; Genentech) were also administered before reperfusion and redosed every 12 hours (Supplemental Figure S1C, http://links.lww.com/HEP/F706). Methylprednisolone (125 mg; Pfizer) was readministered every 8 hours after the initial dosage.

### Procurement of human livers

When human livers acceptable for the study were identified, a team was dispatched to procure livers in a standard manner (Supplemental Methods, http://links.lww.com/HEP/F706).^17^

### Cannulation of explanted human livers

The portal vein, hepatic artery (HA), and infrahepatic vena cava were cannulated with 24–28 Fr (DLP, Medtronic), 10–12 Fr (Bio-Medicus, Medtronic), and 32–40 Fr cannulas (DLP, Medtronic) respectively (Supplemental Figure S1D, http://links.lww.com/HEP/F706). The common bile duct was cannulated with an 8–12 Fr cannula (Bio-Medicus, Medtronic). One liter of cold, isotonic, electrolyte-balanced solution (Normosol, ICU Medical) was flushed through the portal vein and HA to remove preservation solution and prime arterial and venous cannulas. The liver was placed in a sterile isolation bag (Medline) in an organ-preservation chamber in preparation for cross-circulation.

XC between explanted human livers and xeno-support swine: Anesthetic induction and preparation of the xeno-support swine are detailed in Supplemental Methods (http://links.lww.com/HEP/F706). A heparin bolus (30,000 U; Sagent) was administered, and cannulas were placed in bilateral internal jugular veins (19 and 21 Fr; Medtronic) under ultrasound guidance or using open cutdown technique (Supplemental Figure S1E, F, http://links.lww.com/HEP/F706). For livers 1–4, the swine was also cannulated at the right femoral artery (14 Fr; Medtronic) to enable a veno-arterial venous configuration of cross-circulation (Figure 1A). For liver 5, the swine was placed on a veno-venous circuit with the ex vivo liver (Supplemental Figure S6, http://links.lww.com/HEP/F706). Calcium chloride (1 g; Hospira) was administered, and the human liver was connected to the circuit through the portal vein, HA, and vena cava cannulas, thereby marking the start of XC. Perfusion circuit elements consisted of a console (Jostra; Maquet), disposable pump (Rotaflow; Maquet), tubing (Tygon, Saint-Gobain), and venous reservoir (Medtronic) (Supplemental Figure S1A, C, http://links.lww.com/HEP/F706). Pressures (Edwards Lifesciences) and flows (Sonotec) in the portal vein, HA, and vena cava were continuously monitored with a data acquisition system (LabChart, ADInstruments). Veno-arterial flow in the femoral artery return was also continuously monitored and titrated to assist circulatory support of the xeno-support swine. To minimize liver surface desiccation, livers were placed in sterile isolation bags (Medline) when not being assessed or manipulated.

Throughout cross-circulation, swine subjects were maintained on a continuous heparin infusion. Activated clotting time was measured using a whole-blood microcoagulation system (Hemochron; Accriva Diagnostics), and the heparin infusion was titrated to an activated clotting time of 180–250 seconds. Physiological parameters of the swine, including heart rate, electrocardiogram, arterial blood pressure, oxygen saturation (SpO_2_), temperature, and respiratory rate, were continuously monitored and recorded (Supplemental Figure S7A, http://links.lww.com/HEP/F706).

### Tissue sample collection

An atraumatic bowel clamp was placed over the liver edge immediately before excisional biopsy of the clamped tissue using a scalpel. A combination of electrocautery, suture ligation, and prolonged direct pressure was used to achieve biopsy site hemostasis before clamp removal. Tissue samples of the left liver lobe were obtained at 0 hours, 12 hours, and 24 hours of cross-circulation. Right lobe biopsies were omitted due to difficulty maintaining hemostasis, given liver contour and systemic anticoagulation. Tissue processing and staining techniques are provided in Supplementary Methods (http://links.lww.com/HEP/F706).

### Cell counts

Supervised, automated quantification of cells stained by immunohistochemistry and immunofluorescence was performed using open-source image analysis software (ImageJ). Images were color-deconvoluted and converted to grayscale, and intensity thresholds were set at the same level for all images of a particular stain. Particles in the resultant image were counted with the “Analyze Particles” function, with a minimum area set at 20 pixels.

### Bile collection and analysis

Bile was passively drained from the common bile duct cannula into a sterile polypropylene tube containing mineral oil. Every 6 hours, bile volume was measured, and bile chemistries were assessed using a point-of-care hemogas analyzer (epoc, Heska).

### Blinded pathologic review

Pathologic review was performed by a board-certified pathologist subspecialized in liver diseases (VQT). Liver tissue sections were divided into 1 mm^2^ grid. Randomly selected (random.org) grids of tissue were imaged, arbitrarily numbered, and images were delivered to the pathologist without reference to experimental time points or conditions. A standardized acute liver injury scoring rubric was used to evaluate each sample by the degree of microvesicular/vacuolar change, sinusoidal dilatation, hepatocellular congestion, and hepatocyte necrosis (Supplemental Table S3, http://links.lww.com/HEP/F706).

### Serum markers

Blood was collected in sampling tubes (BD Vacutainer) and analyzed by a diagnostic laboratory (Antech Diagnostics) for complete blood count, comprehensive metabolic panel, coagulation panel, and fibrinolytic markers. Serum inflammatory markers were analyzed in duplicate by commercial multiplex cytokine array (Discovery Assay; Eve Technologies).

### Complement lysis

Complement hemolytic activity was quantified using a commercial complement activity (CH50) assay kit (Haemoscan) using the manufacturer’s protocol (Supplemental Methods, http://links.lww.com/HEP/F706).

### Vasoresponsiveness test

The vasoconstrictive effect of selective α_1_-adrenergic receptor activation on the hepatic arterial system was assessed after 24 hours of XC. Phenylephrine (4 mg; West Ward Pharmaceuticals) was administered into the hepatic arterial cannula of ex vivo human livers. Hepatic arterial pressures and flows were continuously recorded.

### Statistics and reproducibility

No data were excluded from the analysis. Student *t* tests and 1-way analysis of variance (with Dunnett or Tukey post hoc test) were performed using statistical analysis software (Prism 8.2.1; GraphPad), and *p* < 0.05 was considered statistically significant.

## RESULTS

### Hemodynamics of xenogeneic cross-circulation

**FIGURE 2:**
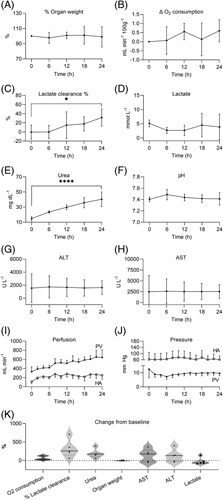
Human liver function over the course of 24 hours of XC. (A) Liver weight is represented as a percentage of the baseline. (B) Change in (Δ) O_2_ consumption from baseline. O_2_ consumption is calculated as the difference between liver inflow and outflow O_2_ content, as computed using the Fick Principle. (C) Lactate clearance, the difference between liver inflow and outflow lactate levels, is represented as a percentage of inflow lactate. (D) Blood lactate concentration. (E) Blood urea concentration. (F) Blood pH. (G) ALT. (H) AST. (I) HA and PV perfusion rates. (J) HA and PV pressures. (K) Changes in functional parameters and liver injury markers between 0 hours and 24 hours of XC. All graphs represent data for human livers (n = 5 independent experiments). All line graph values represent mean ± SD. One-way ANOVA was used to determine statistical significance. Dotted lines on each violin plot (K) show lower and upper quartiles, and the center line represents the median of each sample group. * *p* < 0.05. **** *p* < 0.0001. Abbreviations: ALT, alanine aminotransferase; AST, aspartate aminotransferase; CVF, cobra venom factor; HA, hepatic artery; PV, portal vein; XC, xenogeneic cross-circulation.

Reperfusion of human livers with swine blood marked the initiation of XC (Figure 1). A perfusion strategy akin to those used in liver machine perfusion systems was implemented.^18^ Throughout cross-circulation, the mean portal venous flow was 515 ± 134 mL/min and the mean hepatic arterial flow was 233 ± 77 mL/min. Mean organ inflow pressure at the portal vein was 9.4 ± 2.5 mm Hg and at the HA was 66.5 ± 37.8 mm Hg. Swine remained hemodynamically and biochemically stable over 24 hours of cross-circulation (mean heart rate 102 ± 16 beats/min; systolic blood pressure 97.7 ± 18.7 mm Hg; temperature 37.5 ± 1.0 °C; pH 7.43 ± 0.08; Figure 2F, Supplemental Figure S1, http://links.lww.com/HEP/F706).

### Characteristics of human livers

Human livers included in this study were declined for transplantation due to serologic abnormalities (liver 1), prolonged donor warm ischemia time (livers 2 and 3), steatosis and fibrosis discovered intraoperatively (liver 4), and donor-recipient size mismatch (liver 5). Notably, liver 5 had 19.5 hours of cold ischemia time in static cold storage (Figure 1C). Two livers were procured from donors after brain death and 3 were procured from DCD. Additional clinical characteristics of human liver donors are in Supplemental Table S1 (http://links.lww.com/HEP/F706).

### Functional improvement of human livers

During 24 hours of cross-circulation, liver weight remained stable, suggesting absence of marked cellular swelling or congestive injury (Figure 2A). Oxygen consumption, calculated from inflow and outflow SpO_2_ and partial pressures (Supplemental Methods, http://links.lww.com/HEP/F706), increased from 0.8 ± 0.3 mL/min/100 g to 1.4 ± 0.3 mL/min/100 g, corresponding to a mean increase from baseline of 0.6 mL/min/100 g (*p* = 0.16, Figure 2B). Percentage lactate clearance by the liver, the difference between inflow and outflow lactate as a percentage of inflow lactate, steadily increased over 24 hours from −0.4 ± 15.5% to 31.4 ± 19.0% (*p* = 0.02, Figure 2C), while the arterial lactate of the xeno-support swine remained largely stable (Figure 2D). Serum urea, a marker of hepatic protein metabolism, also steadily increased (*p* = 0.02, Figure 2E). System pH remained physiological between 7.4 and 7.5 without marked fluctuation (Figure 2F). Alanine aminotransferase (Figure 2G) and aspartate aminotransferase (Figure 2H), while elevated at reperfusion, remained stable over the period of cross-circulation, suggesting absence of ongoing hepatocellular injury.

Differential perfusion in the portal vein and HA was maintained (Figure 2I). Ex vivo livers received most of their blood flow (69 ± 3%) from the portal vein and the minority of blood flow (31 ± 3%) from the HA, as is the case in situ.^19^ Total liver inflow was experimentally increased when caval outflow SpO_2_ decreased below 50%. SpO_2_ in the caval outflow was maintained at 55.7 ± 9.2%. HA pressure approximated systemic pressure of the xeno-support swine, while portal vein pressure was maintained under a target of 10 mm Hg (Figure 2J).

Over 24 hours of cross-circulation, liver metabolic activity increased, as evidenced by increased oxygen consumption by 66%, increased absolute lactate clearance (inflow lactate − outflow lactate) by 151%, increased percentage lactate clearance [(inflow lactate – outflow lactate)/inflow lactate] by 322%, and increased urea production by 190% (Figure 2K).

### Multiscale and histopathologic assessment of human livers

**FIGURE 3:**
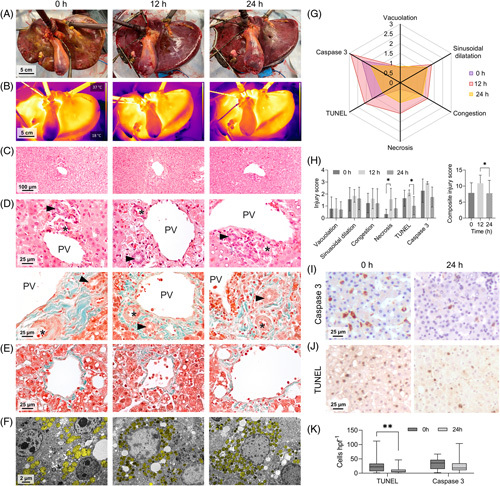
Multiscale analyses of human livers over the course of 24 hours of XC. (A) Gross appearance of livers. (B) Infrared thermography. (C–E) Histologic evaluation of: liver lobule on *H* and *E* staining at low resolution (C), portal triad on *H* and *E* and Trichrome staining at high resolution (PV, *, HA, arrow, bile duct) (D), and central vein on Trichrome staining at high resolution (E). (F) Transmission electron micrographs for visualization of hepatocyte intracellular structures. Mitochondria pseudocolored yellow. (G) Histopathologic liver injury scores visualized using a radar plot. Injury score aligned to the vertical axis represents score in 6 measured categories. Each overlaid radar plot represents an individual time point (n = 5 independent experiments). (H) Categorical and composite liver injury scores. (I–K) Cleaved caspase 3 (I) and TUNEL (J) staining, as well as cell counts (K). All bar graph values represent mean ± SD. Representative images of human livers 1–5 are shown in Supplemental Figures S2–S6. One-way ANOVA (H) and Student *t* test (K) were used to determine statistical significance. Box-and-whisker plot (K) shows lower and upper quartile values, center line represents the median of each sample group, and vertical lines represent maximum and minimum values. * *p* < 0.05. ** *p* < 0.01. Abbreviations: HA, hepatic artery; *H* and *E*, hematoxylin and eosin; HPF, high-power field; PV, portal vein; TUNEL, terminal deoxynucleotidyl transferase dUTP nick end labeling; XC, xenogeneic cross-circulation.

Global appearance and architecture of the human livers remained grossly preserved throughout cross-circulation (Figure 3A). Thermographic imaging indicated uniform perfusion without evidence of regional ischemia (Figure 3B). Histologic evaluation demonstrated minimal necrosis or hepatocellular abnormalities (Figure 3C; Supplemental Figures S2–S3L, M, http://links.lww.com/HEP/F706; Supplemental Figures S4–S5L, N, http://links.lww.com/HEP/F706; Supplemental Figure S6K, M, http://links.lww.com/HEP/F706), maintenance of zonal organization, and integrity of the portal triad (Figure 3D, Supplemental Figures S2–S3N, O, http://links.lww.com/HEP/F706; Supplemental Figures S4–S5M, O, http://links.lww.com/HEP/F706; Supplemental Figure S6L, N, http://links.lww.com/HEP/F706) and central venous structures (Figure 3E). Transmission electron microscopy demonstrated the structural integrity of hepatocytes and abundant mitochondria (Figure 3F).

Liver injury was evaluated by (1) blinded histopathologic assessment by a board-certified liver pathologist, which included quantification of areas of microvesicular or vacuolar changes, sinusoidal dilatation, hepatocellular congestion, and necrosis (Supplemental Table S3, http://links.lww.com/HEP/F706), and (2) (http://links.lww.com/HEP/F706) systematic counting of early (cleaved caspase 3-positive) and late (terminal deoxynucleotidyl transferase dUTP nick end labeling-positive) apoptotic cells. Injury scores were evaluated at 12 hours compared with baseline, likely reflecting reperfusion-associated changes. Injury scores at 24 hours decreased from 12 hours across all injury categories (Figure 3G, H). Mean composite score decreased by 30% from 12 hours to 24 hours [12 h, 10.9 ± 2.6; 24 h, 7.8 ± 4.0; *p* = 0.02, (Figure 3H)], demonstrating significant improvement from reperfusion-related insults over the course of xeno-support.

Comparisons of terminal deoxynucleotidyl transferase dUTP nick end labeling and cleaved caspase 3-positive cells also demonstrated a 57.5% decrease in the number of terminal deoxynucleotidyl transferase dUTP nick end labeling-positive cells (0 h, 26.6 ± 26.7; 24 h, 11.4 ± 12.0; *p* = 0.002; (Figure 3J, K).

### Vascular integrity of human livers and hematologic integrity of cross-circulation system

**FIGURE 4:**
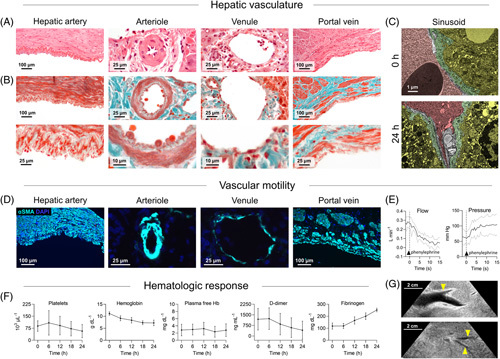
Vascular and hematologic integrity over the course of 24 hours of XC. (A–C) Characterization of hepatic arterial, portal venous, and sinusoidal architecture at end of xenogeneic XC on *H* and *E* staining (A), Trichrome staining (B), and transmission electron microscopy (C), pseudocolored for hepatocyte (yellow), sinusoid endothelial cell (green), and sinusoid space (red). (D) Immunofluorescence of αSMA on hepatic arterial and portal venous vessels. (E) Vasoresponsiveness of HA to phenylephrine after 24 hours of xenogeneic XC. (F) Quantification of platelets and hemolytic injury markers. (G) Characterization of proximal and distal portal venous patency (arrow) at 24 hours of xenogeneic XC (n = 1). All graphs represent data for human livers (n = 5 independent experiments). All line graph values represent mean ± SD. Abbreviations: αSMA, alpha-smooth muscle actin; HA, hepatic artery; *H* and *E*, hematoxylin and eosin; XC, xenogeneic cross-circulation.

Endothelial cells represent the first opportunity for interface between the blood of the xeno-support swine and non–self-antigens in the human liver. Thus, the preservation of endothelial integrity and function is key to maintaining the durability of the XC system. After 24 hours of cross-circulation, large and small HA and portal vein branches retained normal vessel wall organization (Figure 4A, B), endothelial cell morphology (Figure 4B), and absence of microthrombi. Transmission electron microscopy of hepatic sinusoids demonstrated intact endothelial cells at the hepatocyte-sinusoid border (Figure 4C). Surface ultrasound at 24 hours demonstrated patent main and distal branches of the portal vein (Figure 4G).

Immunostaining for α-smooth muscle actin demonstrated myocyte preservation in hepatic vascular smooth muscle (Figure 4D), which is abundant in the arterial tunica media and also present in the wall of the portal vein for endothelial cell support.^20^ The HA demonstrated a rapid decrease in flow and increase in luminal pressure after intra-arterial administration of phenylephrine, indicating preserved α_1_-adrenergic signaling and vasoconstrictive function of the vessel walls (Figure 4E).

Histologic review demonstrated no evidence of thrombosis. Blood platelet count decreased 35.3% from 89 ± 33 10^3^/µL to 57 ± 41 10^3^/µL (*p* = 0.23) but was proportional to the 35.4% decrease in hemoglobin concentration from 11.3 ± 1.4 g/dL to 7.3 ± 0.8 g/dL (*p* = 0.02; (Figure 4F), likely reflecting hemodilution from repeated blood sampling and intravenous infusions. Thrombocytopenia has also been associated with oxygenator and pump components used for perfusion.^21,22^ Plasma-free hemoglobin level remained low, indicating minimal hemolysis. Markers of platelet activation, including fibrinogen and D-dimer, improved throughout 24 hours (Figure 4F). Overall, findings indicate normalization of coagulation and hematologic systems during 24 hours of XC.

### Biliary integrity and cholangiocellular viability during cross-circulation

**FIGURE 5:**
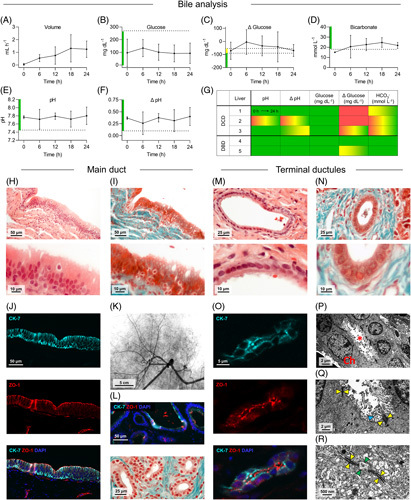
Maintenance of biliary viability over the course of 24 hours of XC. (A–F) Biliary viability markers: bile volume (A), bile glucose (B), bile-blood glucose difference (C), bile bicarbonate (D), bile pH (E), bile-blood pH difference (F). (G) Categorical assessment of each liver at the onset of bile production (left of each cell) and endpoint of cross-circulation (right of each cell) as viable (green), borderline (yellow), or nonviable (red). (H and I; M and N), Histologic assessment of main bile duct on H and E (H) and Trichrome (I) stains, as well as assessment of terminal ductules on H and E (M) and Trichrome (N) stains after 24 hours of xenogeneic XC. (J and K) Integrity of main bile ducts assessed using immunofluorescence for CK-7 and tight junction protein ZO-1 (J), cholangiography (K). (L) Preservation of biliary glands on immunofluorescence for CK-7 and ZO-1, as well as Trichrome staining. (O and P) Terminal ductule integrity assessed using immunofluorescence for CK-7 and tight junction protein ZO-1 (O), as well as transmission electron microscopy (P) showing patent bile duct lumen (*), intact microvilli (blue arrow)(Q), preservation of tight junctions (yellow arrows)—including those around bile canaliculi (green arrows) (R). All graphs represent data for human livers (n = 5 independent experiments). All line graph values represent mean ± SD. Abbreviations: CK, cytokeratin; *H* and *E*, hematoxylin and eosin; XC, xenogeneic cross-circulation; ZO-1, zonula occludens-1.

Post-transplant biliary complications are a frequent cause of morbidity in recipients of high-risk livers.^23,24^ Markers of biliary system health have emerged as important indicators of graft viability on machine perfusion.^8,25^ We evaluated several recently validated cholangiocellular viability criteria: bile pH, bile glucose, bile bicarbonate, difference in bile and perfusate (Δ) pH, and Δ glucose.^8^ Due to differences in perfusate composition between cross-circulation and normothermic machine perfusion systems, the following markers were excluded: perfusate lactate, perfusate pH, and Δbicarbonate. Volume of bile production increased throughout the period of support (Figure 5A). Mean bile glucose remained under 270 mg/dL (Figure 5B) and Δglucose approached −90 mg/dL, indicating improving cholangiocyte ability to uptake and use glucose (Figure 5C). Mean bile bicarbonate increased to above 18 mmol/L at 6 hours and thereafter (Figure 5D). Mean bile pH remained above 7.45 (Figure 5E) and Δ pH remained above 0.10 (Figure 5F), indicating preserved cholangiocyte ability to alkalize bile. Performances of each liver using the 5 recently reported biliary viability criteria are shown in Figure 5G. With the exception of the ΔpH criteria in liver 3, all other viability markers for all livers either improved or remained stable after 24 hours of cross-circulation.

Histologic evaluation demonstrated structural maintenance of biliary architecture in main and intrahepatic bile ducts. After 24 hours of cross-circulation, the main ducts demonstrated columnar biliary epithelium supported by basal connective tissue (Figure 5H, I). Immunostaining demonstrated cholangiocyte expression of cytokeratin (CK)-7 and tight junction protein zonula occludens-1 (ZO-1) (Figure 5J). Cholangiography demonstrated normal biliary anatomy and patency (Figure 5K). Bile duct glands remained continuous with duct lumen and also demonstrated normal histologic architecture, as assessed by CK-7 and ZO-1 staining (Figure 5L). Small ducts demonstrated preserved cuboidal biliary epithelium (Figure 5M, N) with cholangiocytes expressing CK-7 and ZO-1 (Figure 5O). On transmission electron microscopy, biliary architecture seemed intact (Figure 5P), with preservation of cholangiocyte microvilli on the luminal surfaces and tight junctions at apical borders (Figure 5Q). Tight junctions were also preserved between hepatocytes flanking bile canaliculi (Figure 5R).

### Inflammatory response to xenogeneic cross-circulation

**FIGURE 6:**
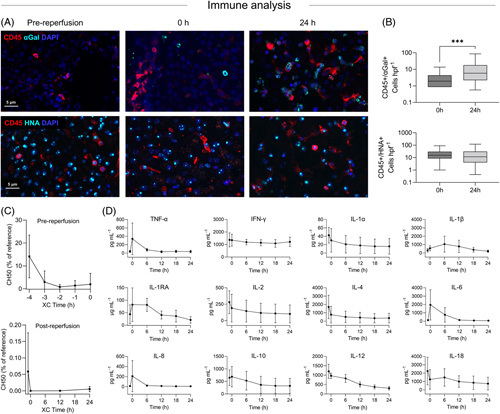
Characterization of immunologic response over the course of XC. (A) Immunofluorescent staining of swine (αGal+) and human (HNA+) leukocyte (CD45+) deposition in the human liver over the course of XC. (B) Quantification of swine and human leukocytes at 0 hours and 24 hours of XC. (C) Quantification of complement activity (CH50) after administration of CVF 4 hours before initiation of XC. (D) Quantification of serum cytokines. All graphs represent data for human livers (n = 5 independent experiments). All line graph values represent mean ± SD. Box-and-whisker plot (B) shows lower and upper quartile values, center line represents the median of each sample group, and vertical lines represent maximum and minimum values. Student *t* test (B) was used to determine statistical significance. *** *p* < 0.001. Abbreviations: CVF, cobra venom factor; HNA, human nuclear antigen; XC, xenogeneic cross-circulation.

Immunostaining for colocalization of the leukocyte marker CD45 and swine surface moiety galactose-α 1,3-galactose (α-Gal) or for colocalization of CD45 and human nuclear antigen enabled identification of swine and human leukocytes, respectively. Similar to xenogeneic studies in lung,^15^ we identified a significant increase in the number of swine CD45-positive cells (0 h, 2.8 ± 2.9; 24 h, 13.4 ± 18.4; *p* = 0.006) but no marked difference in the number of human CD45-positive cells (0 h, 22.7 ± 21.3; 24 h, 23.9 ± 30.7; *p* = 0.8) in the ex vivo liver at 24 hours of XC (Figure 6A, B).

After cobra venom factor administration, CH50 decreased over the ensuing 4 hours before the start of XC (Figure 6C). Complement activity remained suppressed after ex vivo liver reperfusion for the duration of xeno-support.

Multiplex array quantification of porcine serum cytokines demonstrated the greatest fold increase in IL-8 (+15.0-fold), IL-6 (+14.6-fold), and TNF-α (+7.6-fold) at reperfusion, but all decreased throughout XC and approached baseline levels at 24 hours (Figure 6D).

## DISCUSSION

We demonstrate that a XC system can support the viability, histopathologic recovery, and functional rehabilitation of human donor livers declined for transplantation. Livers with steatosis (liver 4), prolonged warm ischemia (livers 2 and 3), and prolonged cold ischemia (livers 1 and 5) consistently achieved functional and histopathologic improvement throughout 24 hours of xeno-support.

Transaminases were elevated from baseline at the initiation of cross-circulation but thereafter plateaued (Figure 2G, H), indicating a transient reperfusion effect. The degree of transaminitis observed was comparable to, if not lower than, those reported in studies of normothermic machine perfusion of human livers.^9,26,27^ Histopathologic injury scoring demonstrated improvement in injury parameters after initial reperfusion (Figure 3G, H), as did decreased concentrations of inflammatory cytokines to baseline levels (Figure 6D).

Livers included in this study had degrees of steatosis, warm ischemia, and cold ischemia that have been shown to increase the risk of primary nonfunction, early allograft dysfunction, and ischemic cholangiopathy.^28^ Despite this, liver function—assessed through oxygen consumption, protein metabolism, and lactate clearance—improved throughout cross-circulation. Oxygen consumption rates in this study were similar to those reported in prior studies of human livers on normothermic machine perfusion.^9,29^

Several parameters for liver viability assessment on machine perfusion have been proposed. Most recently, van Leeuwen et al^8^ reported hepatobiliary viability criteria for livers maintained on normothermic machine perfusion for 2.5 hours. Threshold values of bile volume, perfusate lactate, perfusate pH, bile pH, perfusate-bile pH difference, bile bicarbonate, perfusate-bile bicarbonate difference, bile glucose, and perfusate-bile glucose difference were used to assess livers as viable, borderline, or nonviable. The authors reported that this prediction strategy achieved reduced biliary complications and graft dysfunction in recipients of machine-perfused grafts. After excluding perfusate lactate and pH (which may reflect the physiological state of the xeno-support swine in our study) and bile volume (reported to not be associated with viability or cholangiopathy^27^) as criteria, we applied this viability assessment to the cross-circulation–supported livers in our study. At 0 hours, DCD livers had more criteria in the “nonviable” range compared with the donor after-brain death livers (46.7% vs 0%), consistent with the known increased risk of cholangiopathy associated with DCD, particularly with increased warm ischemia time.^30^ This suggests that the degree of injury sustained by DCD livers used in this study was clinically significant. Although potentially transplantable at the time of procurement, livers 1 and 5 were subject to prolonged cold-ischemic times of 11.5 hours (coupled with the effects of DCD) and 19.5 hours, respectively. Recovery from these substantial ischemic insults highlights the rehabilitative capacity of cross-circulation. Cross-circulation enabled organ recovery such that, at the end of 24 hours of support, all livers demonstrated favorable viability markers (ie, no liver had more than 1 category graded as borderline or nonviable) and seemed to meet criteria suitable for transplantation.

Machine perfusion has grown over the decade as an alternative to static cold storage, particularly for organs more susceptible to ischemia-reperfusion injury. Hypothermic (<12 °C) oxygenated machine perfusion reduces metabolic demand, mitochondrial stress, and generation of oxygen free radicals during the preservation period, as well as decreases ischemia-reperfusion injury, risk of nonanastomotic biliary strictures, and incidence of early allograft dysfunction after transplantation.^31,32^ However, given nonphysiological temperature conditions, Hypothermic oxygenated machine perfusion does not enable accurate viability assessment and is typically used in limited durations of up to 4 hours.^33^

Normothermic (35–37 °C) machine perfusion enables the ex vivo assessment of high-risk donor livers at temperatures that allow physiological hepatic function and metabolism.^8,26,34^ Liver normothermic machine perfusion (NMP) systems have historically been used to support organs ex vivo for up to 12 hours in clinical trials and series.^34,35^ Eshmuminov et al^9^ recently reported their experience using an integrated liver NMP system to support 6 unallocated ex vivo human livers for 7 days. This system included components that managed nitrogenous waste removal (ie, dialysis), glucose homeostasis (ie, insulin and glucagon infusions), metabolic substrates (ie, amino acid and bile salt infusions), simulation of diaphragmatic motion (ie, novel organ bed), and even autoregulation (ie, engineered feedback loops). Despite the impressive composition of this system, it was nevertheless limited in its ability to recover four livers with high-risk clinical features similar to organs used in our study (DCD, warm ischemia time >20 min, and cold ischemia time >4 h). These 4 livers developed severe transaminitis, persistently elevated IL-6 and IL-10 levels, and absent responses to vasoactive agents and pancreatic hormones on NMP.^9^ This is perhaps due to the inability to replicate critical physiological processes necessary to maintaining a milieu that would enable organ rehabilitation and recovery.^36,37^ Such processes include multiorgan synthetic function, neurohormonal signaling, and complex interorgan crosstalk—processes that are preserved with cross-circulation support.

We envision that the multisystem physiological regulation provided through XC can be leveraged to provide whole-biosystem ex vivo liver support. In studies using cross-circulation as a technique for the recovery and rehabilitation of ex vivo donor lungs, our group has demonstrated that this technique enabled the maintenance of ex vivo swine lungs for 4 days,^11^ the recovery of swine lungs severely injured by gastric aspiration,^13^ and the rehabilitation of injured human donor lungs declined for transplantation—including those that had failed ex vivo lung perfusion.^15^

In a parallel effort to increase organ supply, the field of xenotransplantation has endeavored to generate a potentially unlimited pool of organs available for human transplantation. Remarkable progress in biotechnology and genetic engineering has led to the creation of genetically modified swine specifically tailored for human transplantation compatibility.^38^ Recent progress has culminated in landmark efforts to transplant genetically modified swine kidneys and hearts into humans.^4,5^ Despite this momentum in xeno-heart and xeno-kidney transplantation, progress in liver xenotransplantation has been hampered by multifactorial hematologic challenges.^6^ Profound thrombocytopenia, consumptive coagulopathy, and hemorrhagic complications—problems not observed during XC—currently limit liver xenograft survival in swine-to-non–human primate transplantation to under 1 month.^39,40^ In contrast, 435 days and 195-day survival of genetically modified swine-to-non–human primate kidneys and hearts, respectively, had been achieved before the recent swine-to-human xenotransplantation cases.^41,42^

In addition, chronic xenoantigen exposure poses a persistent threat to the long-term durability of xenografts.^43^ Although most xenoreactive responses in hyperacute rejection are targeted to a few carbohydrate antigens that can be addressed through genetic modification, chronic exposure to innumerable species-specific antigens on xenografts can lead to rejection and graft failure.^43–46^ XC can potentially circumvent these challenges. Exposure to swine cells and humoral factors is limited to the duration of cross-circulation. Although we identified swine CD45-positive cells in the human liver during XC, we believe the presence and viability of such xeno-derived cells to be largely limited to the period associated with cross-circulation. On allotransplantation of a human liver recovered by XC, a transient xenoreactive response may occur against deposited swine cells and humoral factors, but the human allograft itself would be expected to be spared persistent xeno-specific reactions. A review of 8 studies found that after exposure to porcine antigens and development of antiporcine xenoantibodies, human recipients of a subsequent kidney or liver allograft did not develop antibody-mediated or accelerated cellular rejection.^47^ These findings suggest that a human organ engrafted with swine xenoantigens during cross-circulation can be accommodated by a human recipient. In addition, circulating swine immune cells and other immune activators can be readily modulated with a variety of existing mechanical and pharmacologic strategies, such as the use of leukocyte filters,^48^ T-cell depletion,^49^ IL-2 pathway antagonists,^50^ and IgG-degrading enzymes.^51^

The limited number, variability in the type and pattern of injury, reason for being declined, and overall quality of the human livers included are inherent limitations to this study. Given organ scarcity and limited numbers of donor livers declined for transplantation (and thus available for research), recruiting large numbers of unallocated human livers and generating comparison groups with similar baseline characteristics were unlikely to be feasible. Thus, this study was designed to enable each liver to serve as its own control for baseline characteristics. Limitations to this study design include inability to compare the rehabilitative effects of cross-circulation with other organ support techniques, such as Hypothermic oxygenated machine perfusion, NMP, and normothermic regional perfusion. Desiccation of the liver surface occurred when organs were uncovered frequently for serial assessment. Active humidification of the organ chamber may decrease capsular desiccation in future studies. A 24-hour timepoint was chosen to establish the feasibility of cross-circulation for the support of human livers—the first report of this application. Viability and improved physiological parameters of each liver at 24 hours suggest that future extended-duration cross-circulation studies may further expand the potential applications of this technique. The complex immunologic milieu in this xenogeneic system was partially characterized in this study, and additional investigation (including effects of human blood re-exposure) is necessary to inform future translational efforts. Furthermore, the transmission of swine-derived pathogens has long been a concern in xenotransplantation.^52^ In the recently reported case of genetically modified swine-to-human cardiac xenotransplantation, porcine cytomegalovirus was identified in the human recipient more than a month after xenograft implantation.^5^ In our cross-circulation system, the risk for zoonotic infection during xeno-support remains to be fully investigated. Pathogen elimination and prevention strategies will be a consideration in future translational studies.

Nevertheless, we envision cross-circulation as a potential donor organ support strategy and scientific research tool. High-risk or extended criteria organs that are declined for transplantation may be recoverable using XC. Critically ill patients with acute liver failure may be candidates for ex vivo support using cross-circulation with declined liver allografts or genetically modified xenografts. As a research tool, cross-circulation may provide unparalleled opportunities for the investigation of biological, pathophysiologic, and immunologic questions, as well as opportunity for extracorporeal liver manipulation and optimization in an intact, physiological system. The milieu provided by XC may be uniquely positioned to support the development of techniques and therapeutics for organ recovery that require, or could be potentiated by, a complete biosystem. Future studies using extended XC for extracorporeal organ maintenance could investigate advanced interventions through chemical conditioning, immunomodulation, viral transfection, cell replacement, or other bioengineering approaches to improve organ function. Ultimately, we envision broad applications for this system as a clinical biotechnology and as a scientific research tool for the development of disease-modifying therapies and strategies that enable organ recovery and rehabilitation.

## AUTHOR CONTRIBUTIONS

Wei Kelly Wu, Rei Ukita, John W. Stokes, Sophoclis P. Alexopoulos, and Matthew Bacchetta: conceptualization. Wei Kelly Wu, Rei Ukita, Yatrik J. Patel, Vincent Q. Trinh, John W. Stokes, Youngmin A. Lee, Jinho Kim, Sophoclis P. Alexopoulos, and Matthew Bacchetta; methodology. Wei Kelly Wu, Rei Ukita, Yatrik J. Patel, Michael Cortelli, Ioannis A. Ziogas, Sean A. Francois, Meredith Mentz, Nancy L. Cardwell, Jennifer R. Talackine, and William M. Grogan: investigation. Wei Kelly Wu, Rei Ukita, Vincent Q. Trinh, Nancy L. Cardwell, and Jennifer R. Talackine: formal analysis. Wei Kelly Wu: visualization. Wei Kelly Wu, Sophoclis P. Alexopoulos, and Matthew Bacchetta: funding acquisition. Wei Kelly Wu, Nancy L. Cardwell, Sophoclis P. Alexopoulos, and Matthew Bacchetta: project administration. Wei Kelly Wu, Rei Ukita, Sophoclis P. Alexopoulos, and Matthew Bacchetta: supervision. Wei Kelly Wu, Sophoclis P. Alexopoulos, and Matthew Bacchetta: writing—original draft. Wei Kelly Wu, Rei Ukita, John W. Stokes, Vincent Q. Trinh, Youngmin A. Lee, Sophoclis P. Alexopoulos, and Matthew Bacchetta: writing—review and editing.

### FUNDING INFORMATION

Burroughs Wellcome Fund Physician-Scientist Institutional Award to Vanderbilt University ID: 1018894 (WKW). H. William Scott, Jr Chair in Surgery Foundation (MB). National Institutes of Health grant HL140231 (MB). Congressionally Directed Medical Research Programs PR212237 (MB).

## ACKNOWLEDGMENTS

The authors thank the following supporters: Vanderbilt Light Surgical Research Laboratory staff J. Diaz, J. Adcock, M.S. Fultz, and M.C. VanRooyen for technical support and research infrastructure; Vanderbilt Translational Pathology Shared Resource staff, including A. Joritz and M. Wilkes for laboratory pathology support (supported by the NCI/NIH Cancer Center Support Grant 5P30CA68485-19); TPSR Research Histology for immunohistochemistry services and use of the Leica Bond RX (supported by the Shared Instrumentation Grant S10OD023475-01A1); Vanderbilt Cell Imaging Shared Resource for transmission electron microscopy processing and imaging services (supported by NIH grants CA68485, DK20593, DK58404, DK59637, and EY08126); Tennessee Donor Services, DCI Inc, for organ research infrastructure; and Vanderbilt transplant coordinators S. Scholl and A. Dutton for procurement administrative support.

### CONFLICTS OF INTEREST

Matthew Bacchetta is a coinventor on a patent application (WO2018013849A1) for cross-circulation as a platform for extracorporeal organ recovery, regeneration, and maintenance. Wei Kelly Wu, John W. Stokes, Rei Ukita, Sophoclis P. Alexopoulos, and Matthew Bacchetta are co-inventors on a patent application for an alternate configuration of cross-circulation for the same purpose. The remaining authors have no conflicts to report.

### DATA AVAILABILITY

All data are available in the main text or the Supplemental Materials, or available from corresponding authors on reasonable request.

## Supplementary Material
