# Supplementary material for: Xenogeneic cross-circulation for physiological support and recovery of ex vivo human livers
Source: Hepatology. 2023 Mar 30;78(3):820–34. doi: 10.1097/HEP.0000000000000357 (PMC10440302; doi:10.1097/HEP.0000000000000357)
Supplement: Supplementary file 1 [file hep-78-820-s001.docx]

**SUPPLEMENTARY INFORMATION**

**Xenogeneic cross-circulation for physiologic support and recovery of *ex vivo* human livers**

W. Kelly Wu, MD^1,2^, Rei Ukita, PhD^2^, Yatrik J. Patel, MD^2^, Michael Cortelli, BS^2^, Vincent Q. Trinh, MD^3^, Ioannis A. Ziogas, MD^1^, Sean A. Francois, MD^2^, Meredith Mentz, BA^2^, Nancy L. Cardwell, BS^2^, Jennifer R. Talackine, MS^2^, William M. Grogan^2^, John W. Stokes, MD^2^, Youngmin A. Lee, MD, PhD^3^, Jinho Kim, PhD^4^, Sophoclis P. Alexopoulos, MD^1^, Matthew Bacchetta MD, MBA^2,5^

^1^ Division of Hepatobiliary Surgery and Liver Transplantation, Vanderbilt University Medical Center, Nashville, TN, USA.

^2^ Department of Cardiac Surgery, Vanderbilt University Medical Center, Nashville, TN, USA.

^3^ Section of Surgical Sciences, Vanderbilt University Medical Center, Nashville, TN, USA.

^4^ Department of Biomedical Engineering, Stevens Institute of Technology, Hoboken, JN, USA

^5^ Department of Biomedical Engineering, Vanderbilt University, Nashville, TN, USA.

**SUPPLEMENTARY FIGURES**


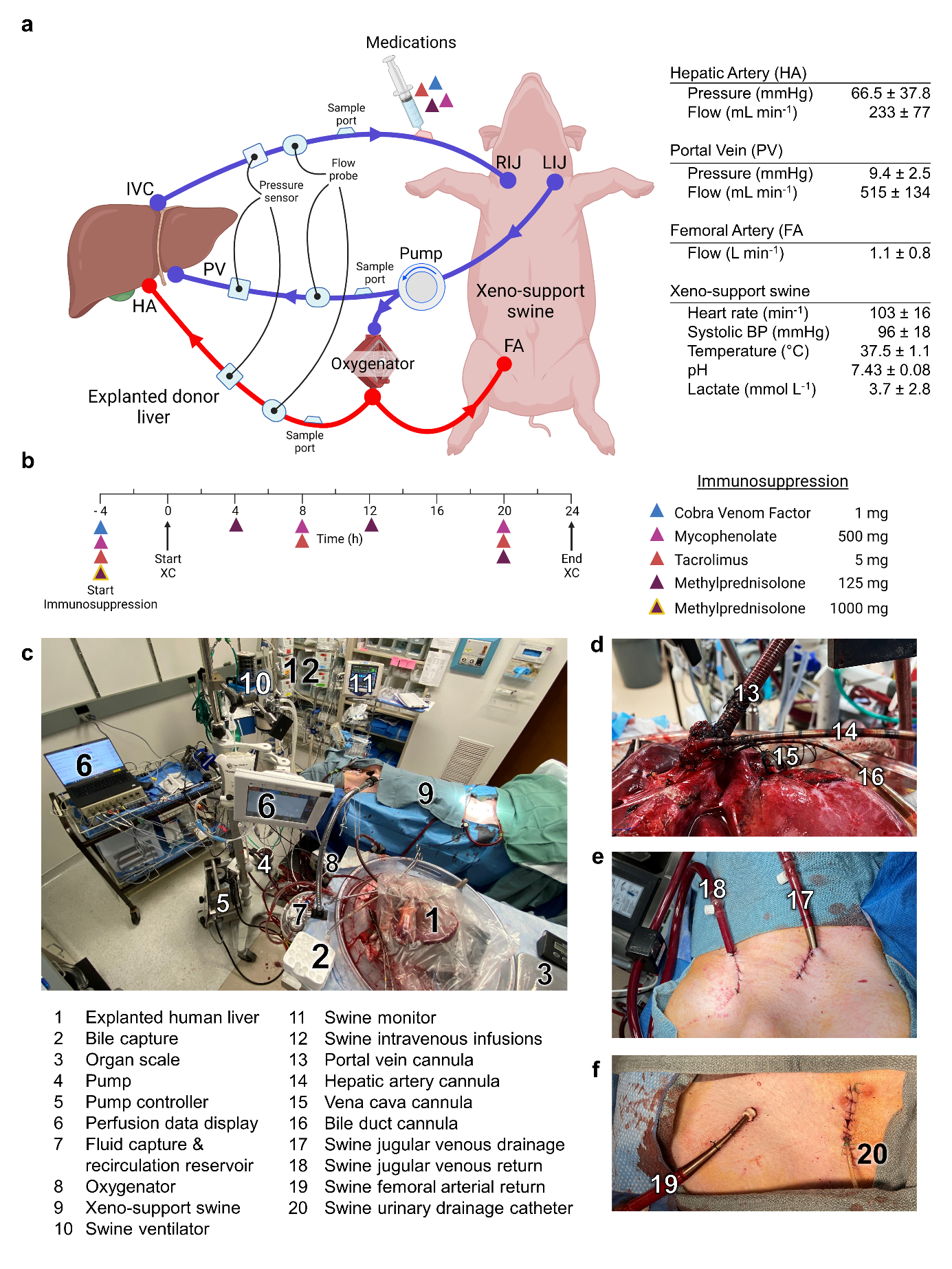


**Fig. S1.** **Experimental setup and xenogeneic liver cross-circulation system parameters.** **a**, Schematic of xenogeneic liver cross-circulation with mean values of perfusion circuit parameters. **b**, Immunosuppression regimen, including induction immunosuppression before cross-circulation and maintenance immunosuppression during cross-circulation. **c**, Experimental setup during xenogeneic liver cross-circulation procedure. **d**, Hepatic artery, portal vein, inferior vena cava, and bile duct cannulas connecting explanted human livers to the xenogeneic cross-circulation circuit. **e**, Xeno-support swine internal jugular (neck) cannulation sites. **f**, Xeno-support swine femoral artery (groin) cannulation site and cystotomy.


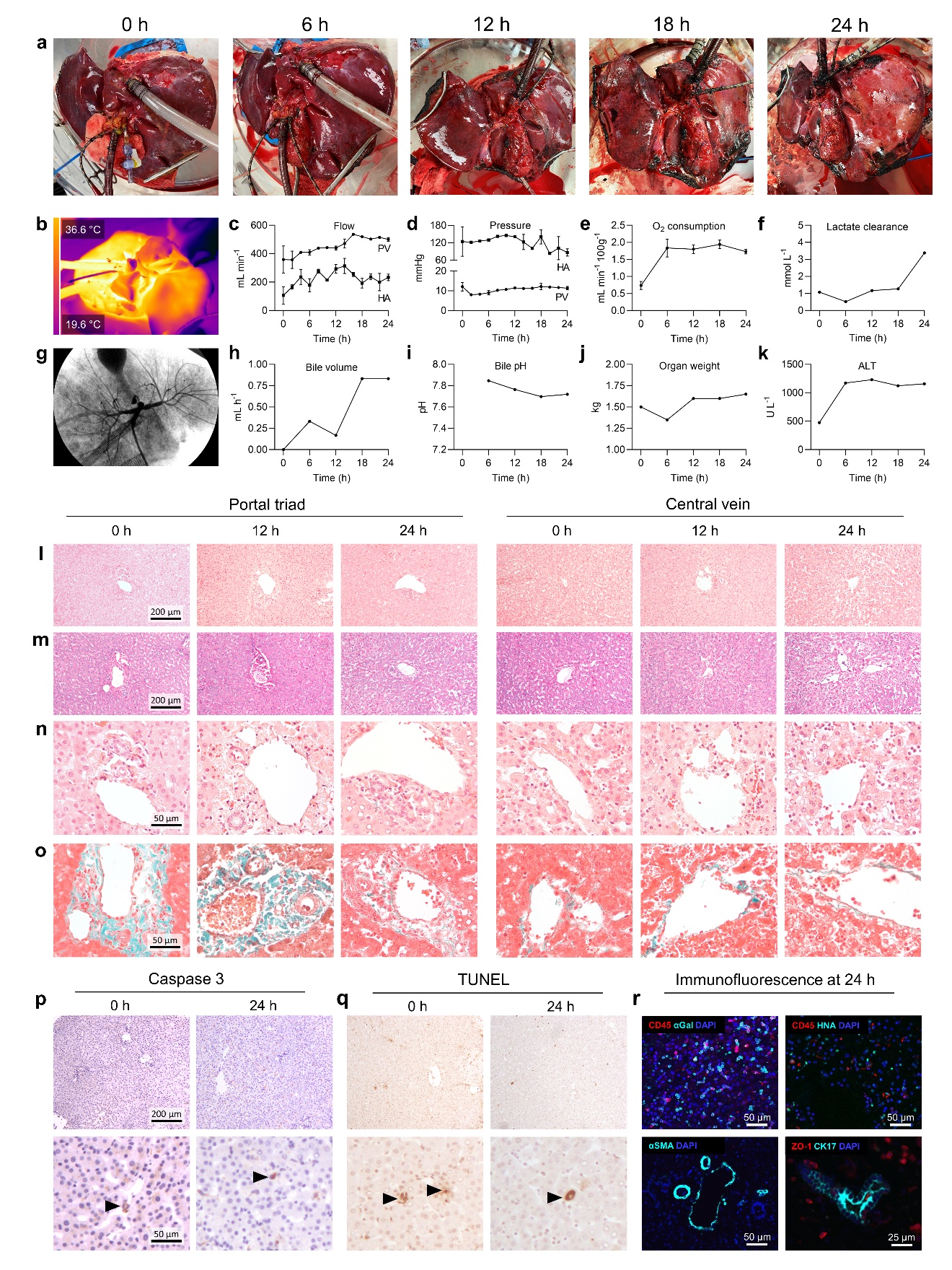


**Fig. S2.** **Multi-scale analyses and functional parameters of human liver 1.** **a**, Gross photography. **b**, Surface thermography at 24 h. **c,** portal vein (PV) and hepatic artery (HA) flow. **d**, PV and HA pressure. **e**, Oxygen consumption. **f,** Lactate clearance. **g,** Cholangiography at 24 h. **h,** Bile volume. **i,** Bile pH. **j,** Liver weight. **k,** Alanine aminotransferase. **l,m,** Liver parenchyma at low magnification on H&E (**l**) and PAS (**m**) staining. **n,o,** Liver parenchyma at high magnification on H&E (**n**) and Trichrome staining (**o**). **p,** Cleaved caspase 3, and **q**, TUNEL staining at 0 h and 24 h. **r**, Immunostaining of swine leukocytes (CD45+/αGal+), human leukocytes (CD45+/HNA), vascular αSMA, and biliary CK-17 and ZO-1 at 24 h. ALT, Alanine aminotransferase. HA, hepatic artery. H&E, hematoxylin and eosin. PAS, Periodic acid–Schiff. PV, portal vein. All graphs represent images and data for human liver 1 (*n* = 1 independent experiment). **c-e**, Data points represent mean ± s.d. of all values obtained at each time point. **f,h-k,** Data points represent a single value obtained at each time point.

**
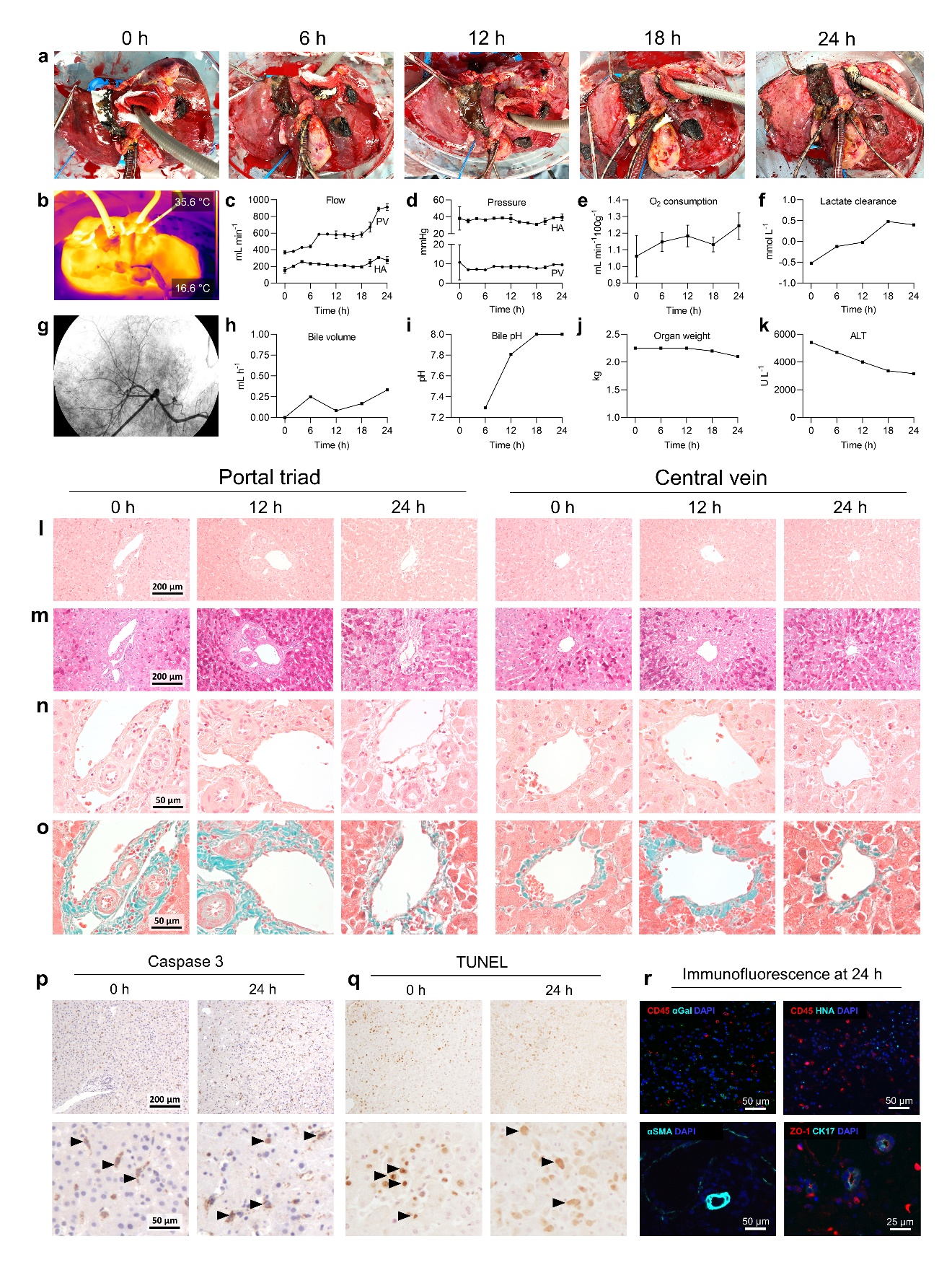
**

**Fig. S3.** **Multi-scale analyses and functional parameters of human liver 2.** **a**, Gross photography. **b**, Surface thermography at 24 h. **c,** portal vein (PV) and hepatic artery (HA) flow. **d**, PV and HA pressure. **e**, Oxygen consumption. **f,** Lactate clearance. **g,** Cholangiography at 24 h. **h,** Bile volume. **i,** Bile pH. **j,** Liver weight. **k,** Alanine aminotransferase. **l,m,** Liver parenchyma at low magnification on H&E (**l**) and PAS (**m**) staining. **n,o,** Liver parenchyma at high magnification on H&E (**n**) and Trichrome staining (**o**). **p,** Cleaved caspase 3, and **q**, TUNEL staining at 0 h and 24 h. **r**, Immunostaining of swine leukocytes (CD45+/αGal+), human leukocytes (CD45+/HNA), vascular αSMA, and biliary CK17 and ZO-1 at 24 h. ALT, Alanine aminotransferase. HA, hepatic artery. H&E, hematoxylin and eosin. PAS, Periodic acid–Schiff. PV, portal vein. All graphs represent images and data for human liver 2 (*n* = 1 independent experiment). **c-e**, Data points represent mean ± s.d. of all values obtained at each time point. **f,h-k,** Data points represent a single value obtained at each time point.


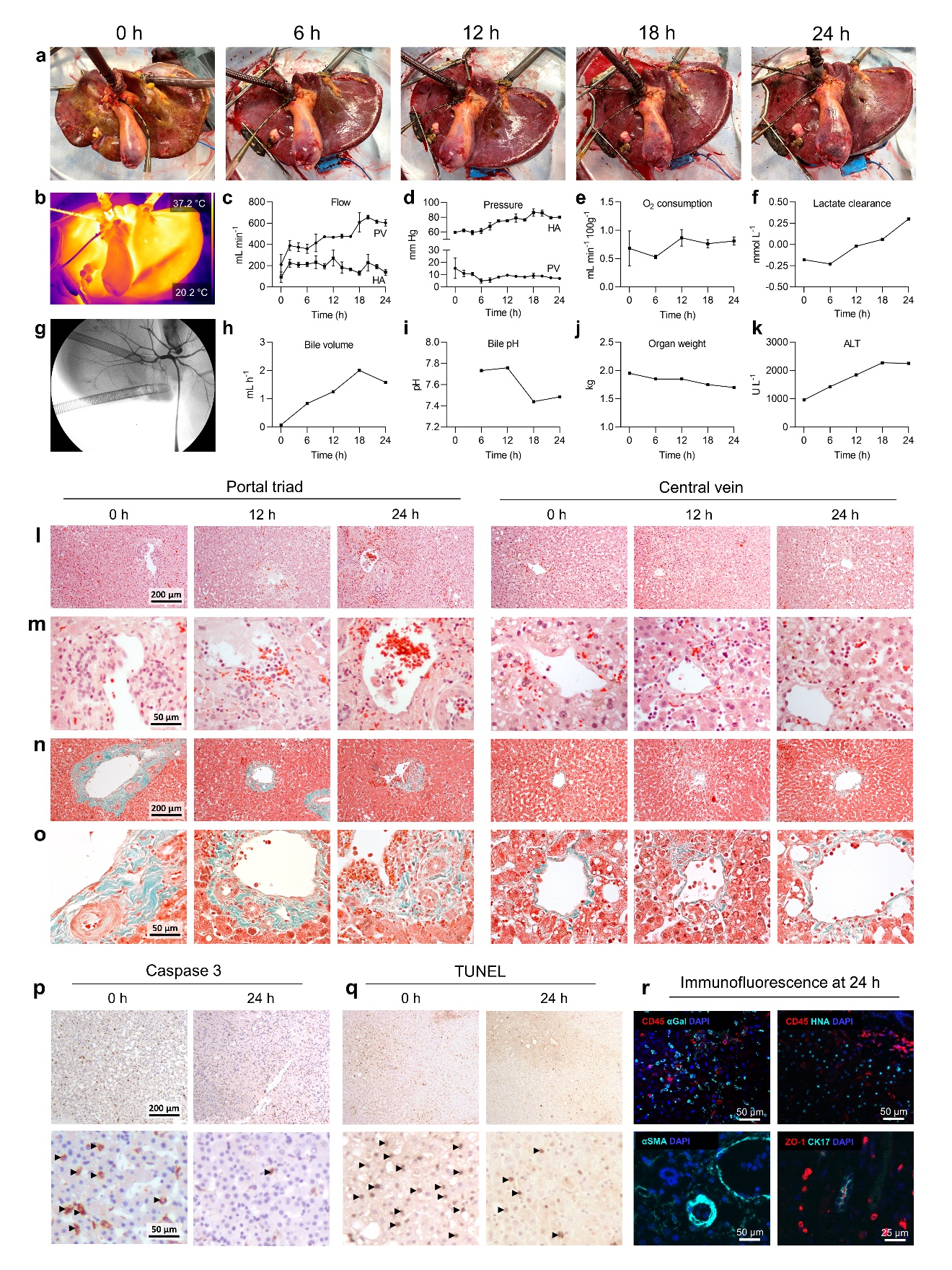


**Fig. S4.** **Multi-scale analyses and functional parameters of human liver 3.** **a**, Gross photography. **b**, Surface thermography at 24 h. **c,** portal vein (PV) and hepatic artery (HA) flow. **d**, PV and HA pressure. **e**, Oxygen consumption. **f,** Lactate clearance. **g,** Cholangiography at 24 h. **h,** Bile volume. **i,** Bile pH. **j,** Liver weight. **k,** Alanine aminotransferase. **l,m,** Liver parenchyma at low (**l**) and high (**m**) magnification on H&E staining. **n,o**, Liver parenchyma at low (**n**) and high (**o**) magnification on Trichrome staining. **p,** Cleaved caspase 3, and **q**, TUNEL staining at 0 h and 24 h. **r**, Immunostaining of swine leukocytes (CD45+/αGal+), human leukocytes (CD45+/HNA), vascular αSMA, and biliary CK17 and ZO-1 at 24 h. ALT, Alanine aminotransferase. HA, hepatic artery. H&E, hematoxylin and eosin. PV, portal vein. All graphs represent images and data for human liver 3 (*n* = 1 independent experiment). **c-e**, Data points represent mean ± s.d. of all values obtained at each time point. **f,h-k,** Data points represent a single value obtained at each time point.


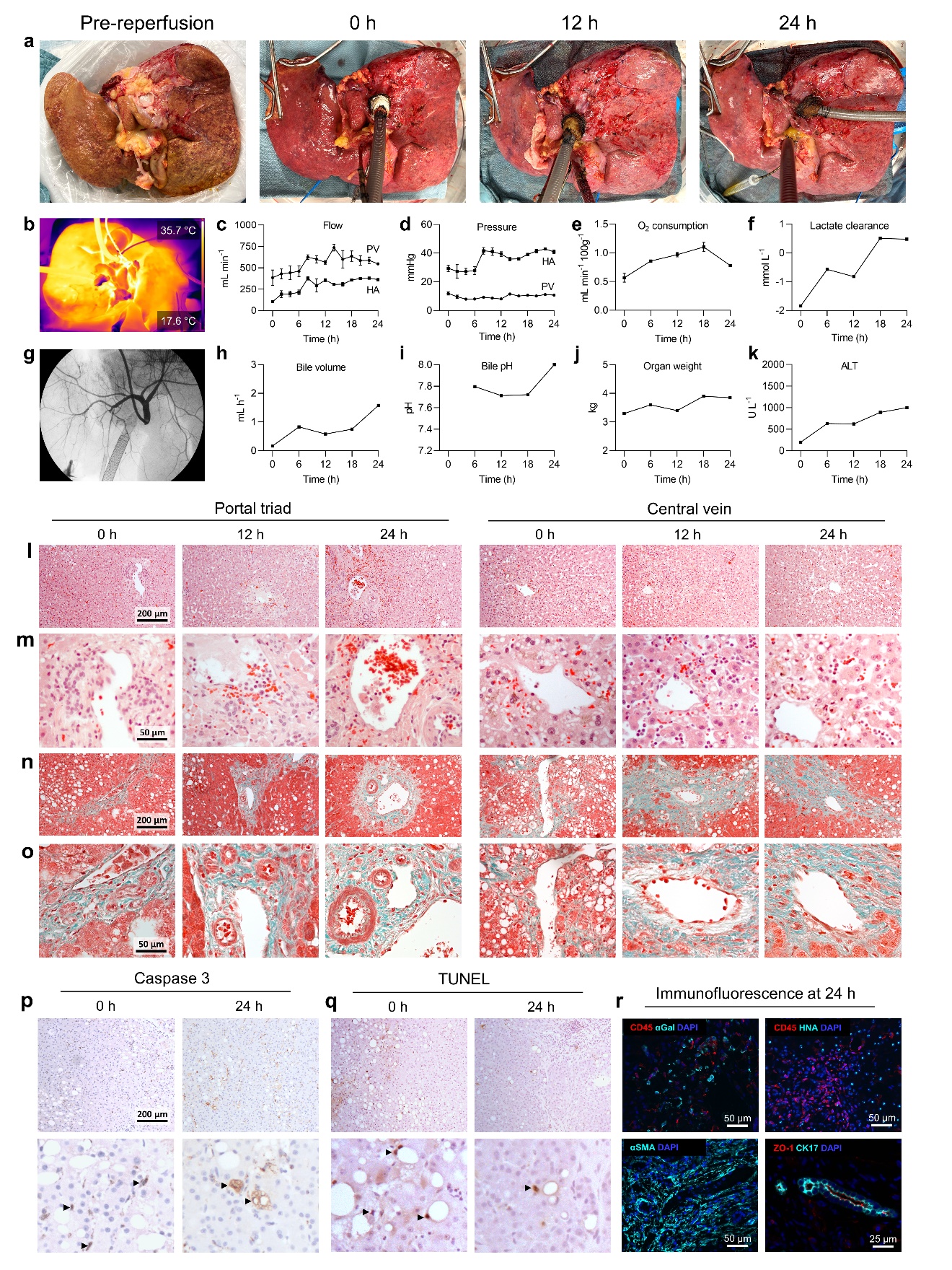


**Fig. S5.** **Multi-scale analyses and functional parameters of human liver 4.** **a**, Gross photography. **b**, Surface thermography at 24 h. **c,** portal vein (PV) and hepatic artery (HA) flow. **d**, PV and HA pressure. **e**, Oxygen consumption. **f,** Lactate clearance. **g,** Cholangiography at 24 h. **h,** Bile volume. **i,** Bile pH. **j,** Liver weight. **k,** Alanine aminotransferase. **l,m,** Liver parenchyma at low (**l**) and high (**m**) magnification on H&E staining. **n,o**, Liver parenchyma at low (**n**) and high (**o**) magnification on Trichrome staining. **p,** Cleaved caspase 3, and **q**, TUNEL staining at 0 h and 24 h. **r**, Immunostaining of swine leukocytes (CD45+/αGal+), human leukocytes (CD45+/HNA), vascular αSMA, and biliary CK17 and ZO-1 at 24 h. ALT, Alanine aminotransferase. HA, hepatic artery. H&E, hematoxylin and eosin. PV, portal vein. All graphs represent images and data for human liver 4 (*n* = 1 independent experiment). **c-e**, Data points represent mean ± s.d. of all values obtained at each time point. **f,h-k,** Data points represent a single value obtained at each time point.


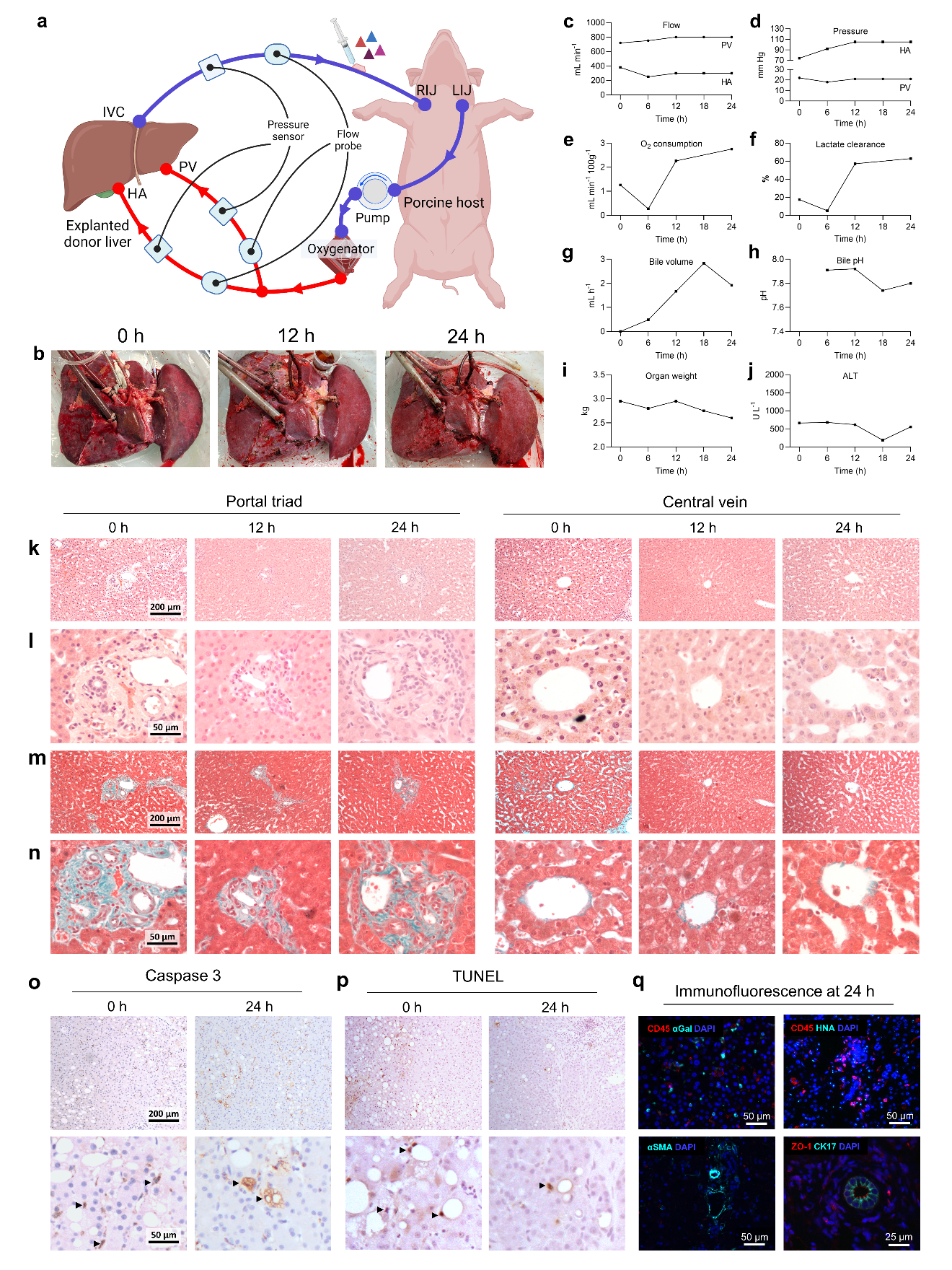


**Fig. S6.** **Circuit schematic, multi-scale analyses and functional parameters of human liver 5.** **a**, Venovenous cross-circulation perfusion circuit used to support human liver 5. **b**, Gross photography. **c,** portal vein (PV) and hepatic artery (HA) flow. **d**, PV and HA pressure. **e**, Oxygen consumption. **f,** Lactate clearance. **g,** Bile volume. **h,** Bile pH. **i,** Liver weight. **j,** Alanine aminotransferase (ALT). **k,l,** Liver parenchyma at low (**k**) and high (**l**) magnification on H&E staining. **m,n**, Liver parenchyma at low (**m**) and high (**n**) magnification on Trichrome staining. **o,** Cleaved caspase 3, and **p**, TUNEL staining at 0 h and 24 h. **q**, Immunostaining of swine leukocytes (CD45+/αGal+), human leukocytes (CD45+/HNA), vascular αSMA, and biliary CK17 and ZO-1 at 24 h. ALT, Alanine aminotransferase. HA, hepatic artery. H&E, hematoxylin and eosin. PV, portal vein. All graphs represent images and data for human liver 5 (*n* = 1 independent experiment). **c-j,** Data points represent a single value obtained at each time point.


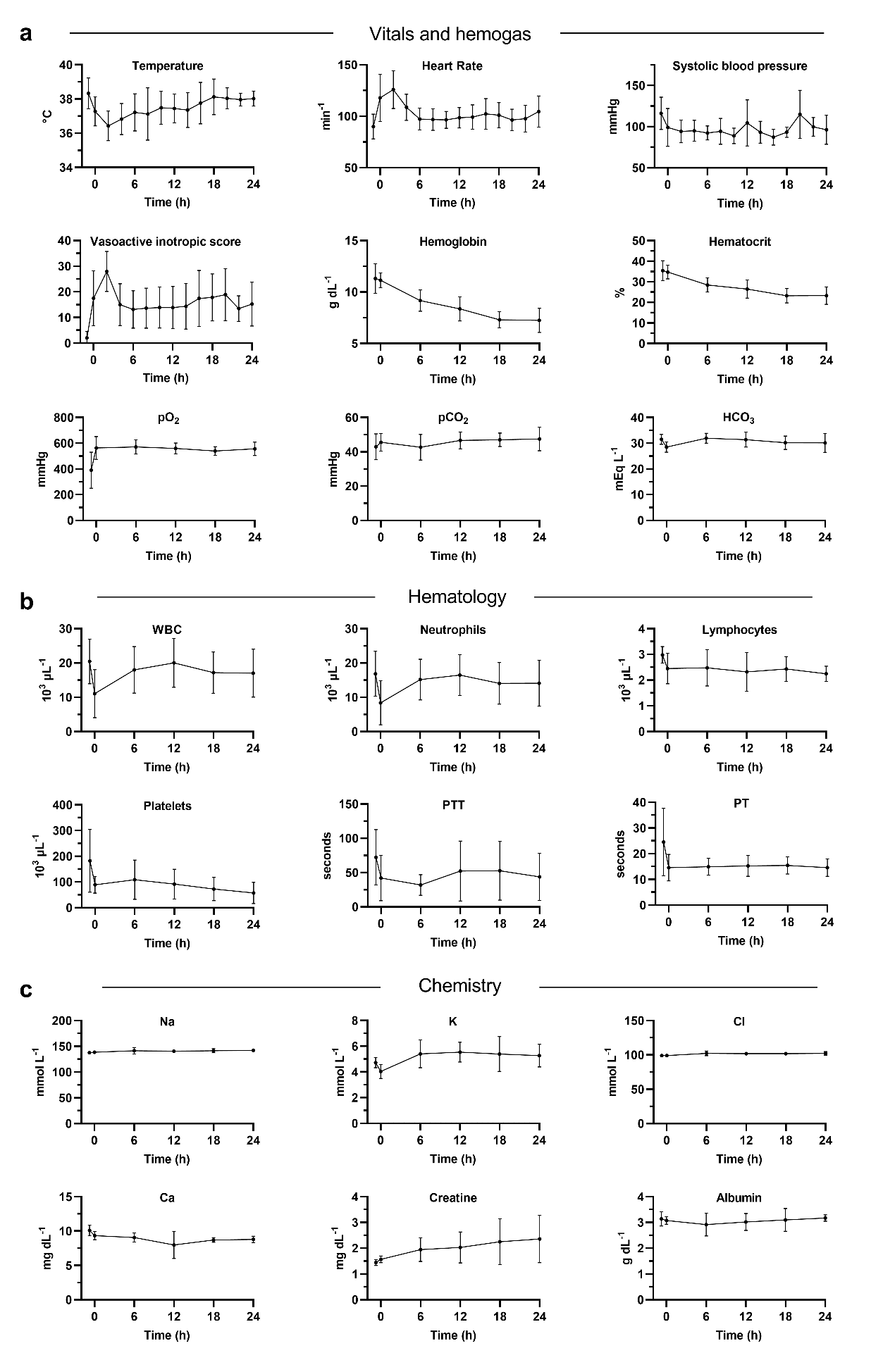


**Fig. S7. Xeno-support swine analyses.** Vitals, hemogas, hematologic, and biochemical evaluation xeno-support swine throughout 24 hours of xenogeneic cross-circulation. All values represent mean ± s.d. (n = 5 independent experiments). WBC, white blood cell. PTT, partial thromboplastin time. PT, prothrombin time.

**SUPPLEMENTARY TABLES**

| **CHARACTERISTIC** | **DONOR 1** | **DONOR 2** | **DONOR 3** | **DONOR 4** | **DONOR 5** |
| --- | --- | --- | --- | --- | --- |
| **Demographics** |  |  |  |  |  |
| Age (years) | 14 | 20 | 34 | 37 | 47 |
| Sex | Male | Male | Male | Male | Male |
| Weight (kg) | 62 | 68 | 92 | 120 | 86 |
| **Medical history** |  |  |  |  |  |
| Liver disease | None | None | None | Significant alcohol use | None |
| Liver biopsy findings | N/A | N/A | N/A | 30% macro-steatosis | No steatosis, normal parenchyma |
| Peak bilirubin (g dL^-1^) | 1.3 | 0.7 | 0.9 | 1.0 | 0.8 |
| **Donation** |  |  |  |  |  |
| Donor type | DCD | DCD | DCD | DBD | DBD |
| Cause of death | Traumatic brain injury | Polytrauma, traumatic brain injury | Polytrauma, traumatic brain injury | Hemorrhagic stroke | Drug intoxication |
| Time from brain death to procurement (h) | -- | -- | -- | 46.5 | 49.6 |
| Warm ischemia time (min)* | 21 | 41 | 37 | -- | -- |
| Cold ischemia time (h) | 11.8 | 12.1 | 9.1 | 6.4 | 19.5 |

**Table S1.** Demographic and clinical characteristics of human liver donors. * defined as the interval from time of agonal phase (donor systolic blood pressure less than 80 mm Hg, or donor oxygen saturation less than 80%) to initiation of core cooling.

|  | **Host species** | **Vendor** | **Product number** | **Clone name** | **Incubation time (min)** | **Dilution** |
| --- | --- | --- | --- | --- | --- | --- |
| **Primary antibody** |  |  |  |  |  |  |
| Anti-cleaved Caspase-3 | Rabbit | Cell Signaling Technology | 9664 | 5A1E | 60 | 1:300 |
| Anti-CD45 | Rabbit | Abcam | ab10559 | N/A | 60 | 1:200 |
| Anti-galactose-alpha-1,3-galactose | Mouse | LS Bio | LS-C63415 | M86 | 60 | 1:5 |
| Anti-human nuclear antigen | Mouse | Abcam | ab190710 | NM95 | 60 | 1:50 |
| Anti-alpha smooth muscle actin | Mouse | ThermoFisher | MA1-06110 | 1A4 | 60 | 1:400 |
| Anti-ZO-1 | Rabbit | Abcam | ab216880 | N/A | 60 | 1:100 |
| Anti-cytokeratin 7 | Mouse | Abcam | ab9021 | RCK105 | 60 | 1:75 |
| **Secondary antibody** |  |  |  |  |  |  |
| Anti-Mouse (647nm) | Goat | Abcam | ab150115 | N/A | 60 | 1:200 |
| Anti-Rabbit (568nm) | Goat | Abcam | ab175471 | N/A | 60 | 1:200 |

**Table S2.** Primary and secondary antibodies.

| **Injury score** | **0** | **1** | **2** | **3** |
| --- | --- | --- | --- | --- |
| Microvesicular/vacuolar changes | <5% | 5 – 33% | 34 – 66% | >66% |
| Sinusoidal dilatation | None | Rare/focal | Patchy, <50% of the slide | >50% of the slide |
| Hepatocellular congestion | None | Rare/focal | Patchy, <50% of the slide | >50% of the slide |
| Hepatocellular necrosis | None | Rare/focal dropout | <33% of the slide | 33-66% of the slide |
| TUNEL (average of 4 hpf) | <5 | 5 – 15 | 15 – 30 | >30 |
| Cleaved Caspase 3 (average of 4 hpf) | <5 | 5 – 15 | 15 – 30 | >30 |

**Table S3.** Scoring rubric of acute liver injury score. hpf: high-power field. TUNEL: Terminal deoxynucleotidyl transferase dUTP nick end labeling.

**SUPPLEMENTARY METHODS**

*Oxygen consumption calculation*

Percent oxygen saturation (SaO_2_), partial pressure of oxygen (PaO_2_), and hemoglobin (Hb) levels were measured in blood drawn from the portal vein (PV) cannula and from the inferior vena cava (IVC) cannula using a point-of care blood analysis system (epoc, Heska). Blood flow (Q) in the hepatic artery (HA), PV, and IVC cannulas are measured. Because HA blood derives directly from the oxygenator (**Fig. 1a**), the SaO_2,HA_ is assumed to be 100%. Oxygen consumption was calculated using the equation below.

$${Total organ O}_{2} consumption \left( {mL min}^{-1} \right)=Q_{PV} \times\left( 1.34\times Hb\times\frac{SaO_{2, PV}}{100}+0.0032 \times PaO_{2,PV} \right)+Q_{HA} \times\left( 1.34\times Hb \right)-Q_{IVC}(1.34\times Hb\times\frac{SaO_{2, IVC}}{100}+0.0032 \times PaO_{2,IVC})]$$

Where,

*Q_PV_ =* PV blood flow (dL min^-1^)

*Q_HA_* = HA blood flow (dL min^-1^)

*Q_IVC_* = IVC blood flow (dL min^-1^)

*SaO_2,PV_* = PV oxygen saturation (%)

*SaO_2,IVC_* = IVC oxygen saturation (%)

*PaO_2,PV_* = PV partial pressure of oxygen (mm Hg)

*PaO_2,IVC_* = IVC partial pressure of oxygen (mm Hg)

*Hb* = hemoglobin level (g dL^-1^)

Given differences in liver weight across experiments, oxygen consumption was normalized to every 100 g of organ weight in **Fig. 2b**.

*Lactate clearance calculation*

Pre- and post- (designated as described in *Oxygen consumption calculation* above) organ lactate levels were measured using a point-of care blood analysis system (epoc; Heska). Percent lactate clearance was calculated using the equation below.

$$lactate clearance (\%)= \frac{{lactate}_{post}-{lactate}_{pre}}{{lactate}_{pre}}$$

Where,

${lactate}_{pre}=$ pre-organ lactate level (mmol L^-1^)

${lactate}_{post}=$post-organ lactate level (mmol L^-1^)

*Anesthetic induction and preparation*

All swine xeno-support subjects (n=5) underwent intramuscular anesthetic induction with ketamine (2.2 mg/kg; Zoetis), tiletamine (4.4 mg/kg; Zoetis), and xylazine (2.2 mg/kg; Bimeda). Anesthesia was maintained with continuous intravenous infusions of fentanyl citrate (0.03-0.1 mg/kg/h; Hospira) and inhaled isoflurane (1–3% in oxygen; Henry Schein). Cefazolin (20 mg/kg; West Ward Pharmaceuticals) and enrofloxacin (5 mg/kg; Norbrook) were administered before skin incision, and re-administered every 8 hours and 24 hours, respectively. An auricular or femoral arterial line (Arrow International) was placed for hemodynamic monitoring and periodic arterial sampling. The immunosuppression regimen was then administered.

*Procurement of human livers*

All liver procurements were performed in coordination with other teams deployed for organ procurement (e.g., heart, lung, kidney) for clinical transplantation. At the time of procurement, a bolus of heparin (30,000 U) was administered intravenously, the aorta was clamped, and cold organ preservation solution (HTK, Custodiol) was administered through aorta and inferior mesenteric vein cannulas. Livers were topically cooled with sterile ice slush *in situ*, then explanted and placed in a sterile isolation bag with 500 ml of organ preservation solution (HTK, Custodiol) at 4 °C. The bag was placed in a second sterile isolation bag containing ice slush and transported on ice to our research facility.

*Tissue processing*

Specimens were immediately fixed in 10% formalin for 24 to 72 hours, embedded in paraffin and sectioned at 5-μm thickness. Sections were stained with hematoxylin and eosin, Trichrome, periodic acid-Schiff, or appropriate antibodies for immunohistochemistry, and then examined under light microscopy.

*Immunohistochemical staining*

Slides were placed on the Leica Bond Max immunohistochemistry stainer. Human liver sections were de-paraffinized and incubated in EDTA-based, pH 9 buffer (Epitope Retrieval Solution 2, BOND) for antigen retrieval for 15 minutes, then placed in a protein block (DAKO) for 10 minutes. Primary antibodies were added and incubated for the duration listed in **Table S2**. Secondary antibodies, if used, were diluted 1:200 and incubated for the duration listed in **Table S2.**

*Transmission electron microscopy*

Liver tissue samples were fixed with 2% glutaraldehyde, 2% paraformaldehyde in 0.1 M sodium cacodylate buffer (pH 7.4). Samples were then post-fixed sequentially in 1% tannic acid, 1% OsO4, and stained en block in 1% uranyl acetate. Samples were dehydrated in ethanol and embedded (EMbed-812, Electron Microscopy Sciences). Sections with nominal thickness of 70 nm were prepared using a ultramicrotome (UC7, Leica), stained with uranyl acetate and lead citrate, and imaged with a transmission electron microscope (TECNAI T12, FEI) at 100 kV. Micrographs were captured with a CMOS camera (NanoSprint, AMT) and recorded (Image Capture Engine, version 7.01.290, AMT).

*Complement lysis*

In brief, 50 μL of plasma samples were serially diluted to 4, 8, 16, 32, 64, and 128 times and incubated with 50 μL of ovine erythrocyte suspension for 30 min at 37 °C. 100 μL of stop solution was added to all tubes, and all samples were centrifuged at 400 × g for 10 min. Supernatants (100 μL) were collected and transferred to 96-well microplates. Optical density at 415 nm was measured (Synergy HTX, BioTek). CH50 was calculated as per manufacturer’s instructions and normalized to normal human serum.
